# Detection of respiration-induced field modulations in fMRI: A concurrent and navigator-free approach

**DOI:** 10.1162/imag_a_00091

**Published:** 2024-02-12

**Authors:** Alexander Jaffray, Christian Kames, Michelle Medina, Christina Graf, Adam Clansey, Alexander Rauscher

**Affiliations:** Department of Physics and Astronomy, University of British Columbia, Vancouver, Canada; Department of Pediatrics, University of British Columbia, Vancouver, Canada; Department of Mechanical Engineering, University of British Columbia, Vancouver, Canada; Department of Radiology, University of British Columbia, Vancouver, Canada

**Keywords:** fMRI, physiological noise, off-resonance, field mapping, respiratory monitoring, quantitative susceptibility mapping

## Abstract

Functional Magnetic Resonance Imaging (fMRI) is typically acquired using gradient-echo sequences with a long echo time at high temporal resolution. Gradient-echo sequences inherently encode information about the magnetic field in the often discarded image phase. We demonstrate a method for processing the phase of reconstructed fMRI data to isolate temporal fluctuations in the harmonic fields associated with respiration by solving a blind source separation problem. The fMRI-derived field fluctuations are shown to be in strong agreement with breathing belt data acquired during the same scan. This work presents a concurrent, hardware-free measurement of respiration-induced field fluctuations, providing a respiratory regressor for fMRI analysis which is independent of local contrast changes, and with potential applications in image reconstruction and fMRI analysis.

## 1 Introduction

Functional magnetic resonance imaging (fMRI) images time-varying fluctuations in MRI signal associated with changes in blood oxygenation and blood oxygenation level dependent (BOLD) contrast (Ogawa et al., 1992). It is used to identify and map regions of increased and decreased blood oxygenation across the brain, which have been correlated with brain function (Ogawa et al., 2000). Its popularity has led it to become commonplace in medical imaging research investigating brain function at rest and in response to stimulus, under a variety of conditions and states. For BOLD fMRI, data are usually acquired with a T_2_*-weighted single-shot echo-planar scan. Neural activity causes a local increase in cerebral blood flow (CBF). Due to the resulting increase in venous blood oxygenation, veins become less paramagnetic and the field inhomogeneities around the vessels decrease (Rauscher et al., 2005). Consequently, the loss of signal coherence in the vicinity of blood vessels is slowed down, resulting in an increase in the T_2_* weighted signal (Ogawa et al., 1992). Changes in CBF are not only due to neural activity, for example blood CO_2_ acts as a strong vasodilator. Adding 5% CO_2_ to the inhaled air or oxygen causes an increase in CBF by around 50% (Rauscher et al., 2005). With the brain’s oxygen demand remaining unchanged, the venous blood oxygenation increases, even in the absence of neural activity.

Fluctuations in blood CO_2_ also occur due to variations in the depth and frequency of breathing, which have a direct influence on cerebral blood flow and consequently influence the BOLD signal in the fMRI scan (Chang & Glover, 2009). A breath hold of 30 seconds, for instance, was shown to increase the BOLD signal by 3-5% (Kastrup et al., 1999). Regular respiratory frequency is around 0.2 to 0.4 Hz, with deep breaths occurring at a rate of 0.05-0.15 Hz (Power et al., 2020). Low-frequency fluctuations are particularly problematic in resting-state fMRI (rs-fMRI), as this technique infers functional connections within the brain from correlations in low-frequency fluctuations in the BOLD signal, which are in the range of 0.1 Hz (Biswal et al., 1995). End tidal fluctuations in CO_2_ occur at around 0.05 Hz and are correlated with BOLD fMRI signal fluctuations (Wise et al., 2004). Birn et al. found that respiration depth varied by 19.1% ± 8.6% during a lexical task and by 17.9% ± 4.2% when subjects were cued to breath at a constant rate and these changes were significantly correlated to BOLD signal changes (Birn et al., 2006).

Respiratory information can be used to model respiratory contributions to the fMRI signal. Regressing out breathing fluctuations during fMRI analysis requires a respiratory measure. External monitoring of respiration using a breathing belt remains the gold standard, and robust methods have been developed to use the measurements from such sensors to remove noise from fMRI acquisitions (Glover et al., 2000; Kasper et al., 2017). Power et al. analyzed 440 rs-fMRI scans from the Human Connectome Project data set and found a wide variety of intra- and inter-subject breathing periodicity (Power et al., 2020; Van Essen et al., 2013). They observed that over the 14.4 minutes scan, respiratory rate decreased along with depth of breathing, whereas the variance increased (Power et al., 2020).

Single-shot gradient echo (GRE) echo-planar imaging (EPI) sequences with low spatial resolution and high temporal resolution are commonly used for fMRI (Glover, 2011; Ogawa et al., 1992). The signal evolution in a GRE sequence can be described by a magnitude which changes according to the aforementioned tissue-specific decay constant T_2_*, as well as a phase which accumulates with TE and scales with the local resonant frequency offset from the main magnet field B_0_ (Rauscher et al., 2006; Reichenbach & Haacke, 2011). The local field offset is determined by the distribution of magnetic susceptibility within and outside of the reconstructed image, and as such, GRE sequences are widely used as input to processing techniques including quantitative susceptibility mapping (QSM) and susceptibility weighted imaging (SWI) (Rauscher et al., 2006, 2011; Reichenbach & Haacke, 2011; Wang & Liu, 2015). These image processing techniques use the information about the local magnetic field encoded in the image phase to quantify the local susceptibility distribution and enhance the depiction of vasculature (Ayaz et al., 2011; Reichenbach et al., 1997).

Considering the phase, each reconstructed fMRI volume is a complex valued depiction of the magnetization sampled around the echo time (TE). It has been shown that respiration induces changes in the magnetic field inside the brain of 2-5 Hz (which is ~0.01 ppm), contributing to physiological noise in the fMRI measurement (Van de Moortele et al., 2002; Vannesjo et al., 2018). This field fluctuation amplitude is within the sensitivity range of phase processing routines employed in QSM (Deistung et al., 2017). Local changes in susceptibility have been observed by processing the phase of fMRI acquisitions in an approach known as functional QSM, an emerging technique which provides motivation for the observability of small field fluctuations in the fMRI scan (Sun et al., 2017). While QSM is sensitive to changes in local susceptibility, the measured phase contains contributions from both the local tissue fields and the harmonic background field, which can confound the measurement of susceptibility values in the volume of interest. It is necessary in QSM to subtract the background field to isolate the local susceptibility distribution (Sun & Wilman, 2014). One of the useful properties of the background field is that it is harmonic and therefore does not contain contributions from signals within the imaging volume. For the fMRI scan, this means that any temporal changes in the harmonic field must comprise field changes which are independent of the BOLD signal.

Although temporal fluctuations in the harmonic field are independent of BOLD contrast, they cannot be attributed to a specific source of physiological noise without additional constraints. In this work, the identification of harmonic field fluctuations which result from respiratory motion is approached using a blind source separation (BSS) strategy (Golub & Reinsch, 1970). BSS attempts to isolate signals from multiple sources which have been measured with a single channel. In the case of respiratory field estimation, the fluctuations in the background field are a superposition of a set of fields arising from different susceptibility distributions in the imaging environment and their behavior.

We can model susceptibility sources as dipoles, which have a magnetic field which decays with the cube of the distance from the dipole $B\left(r\right)\propto \frac{1}{r^{3}}$, and so any harmonic field existing within the imaging volume is attributable to nearby susceptibility sources. In the case of fMRI and neuroimaging, this is the body tissue, which is relatively static during the imaging protocol and finite in extent, with motion that can be well-described by a small number of terms (Welch et al., 2002). Using a truncated singular value decomposition (SVD), a small set of spatial basis functions and time-varying coefficient vectors can be found which approximate the spatiotemporal evolution of the harmonic field.

We propose to process the phase of fMRI sequences to capture respiration-induced changes in the harmonic field within the imaging volume. The time-course of the harmonic field is approximated using SVD to separate breathing-induced field modulations from other sources of noise (Agrawal et al., 2020). The resulting field modulation can be further projected onto the set of solid harmonic basis functions, which are used to describe the field fluctuations during acquisition that stem from respiration.

## 2 Methods

We acquired a set of 10 fMRI scans in 7 healthy volunteers (weight: 79.7 ± 12.3 kg, height: 1.80 ± 0.08 m) at 3 T (Philips Elition) using a gradient-echo EPI sequence (duration ~5 minutes) and TR/TE of 1150/30 ms. An isotropic voxel size of 2.5 mm, a parallel imaging factor of 2, and a multi-band factor of 3 (overall R = 6) were employed to achieve a short TR (Pruessmann et al., 1999). Complex data were exported at the console. The size of the reconstructed data was 96 × 96 × 57 × 260 corresponding to 3 spatial dimensions and one temporal dimension respectively. No zero-filling or partial Fourier acquisition was performed. Nine (9) of the scans were acquired during the imaging portion of a study on concussion in ice hockey players, and a control subject was acquired separately. Three of the subjects were scanned twice. The study was approved by the University of British Columbia Clinical Research Ethics Board (H21-00400). All volunteers gave written, informed consent.

Respiration of the subjects was monitored using the scanner's respiratory physiology sensor (500 Hz sampling rate, positioned around lower chest). The control subject was instructed to perform a series of controlled breathing patterns during the scan: A) 10 short breaths B) 10 deep breaths, C) free-breathing, D) breath-hold of 10 seconds to 20 seconds. All other subjects were given no specific instructions regarding respiration.

**Fig. 1.**
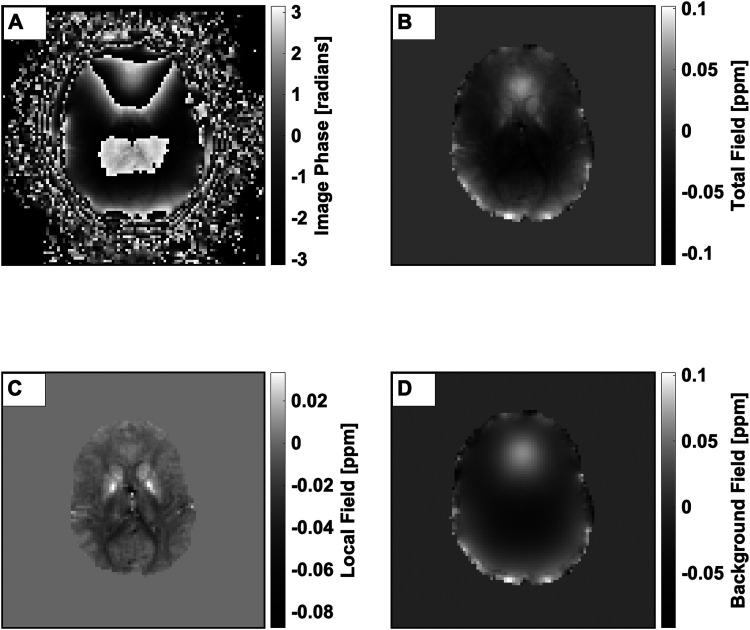
Illustration of the pipeline from raw image phase to harmonic background field. All images are of a single slice taken from the first timepoint of the fMRI time-series. (A) Raw image phase. (B) Image phase after unwrapping with Laplacian unwrapping. (C) The projection of the total field onto the local susceptibility sources within the volume of interest. Note the absence of low-frequency phase modulation across the slice. (D) The isolated harmonic field, obtained by subtraction of the local field in (C) from the total field depicted in (B).

After acquisition, the resting-state fMRI data were sliced along the temporal dimension to produce 260 volumes which were individually processed (Fig. 1) using the following steps: Laplacian-based phase unwrapping was performed to remove phase discontinuities in the image (Schofield & Zhu, 2003). The local field was estimated from the unwrapped phase with regularized SHARP (Sun & Wilman, 2014). Then, the background harmonic field was estimated by subtracting the local field from the total field.

The estimated harmonic field data from all volumes was reshaped into a M×N matrix P where M is the number of spatial voxels and N is the number of time samples during the acquisition. The field was then decomposed using Singular-Value Decomposition (SVD) and a temporally low-rank representation of the background field was reconstructed by selecting elements on the diagonal of the matrix *S* to obtain $S_{L}$ (Algorithm 1) (Wold et al., 1987).

**Fig. 2.**
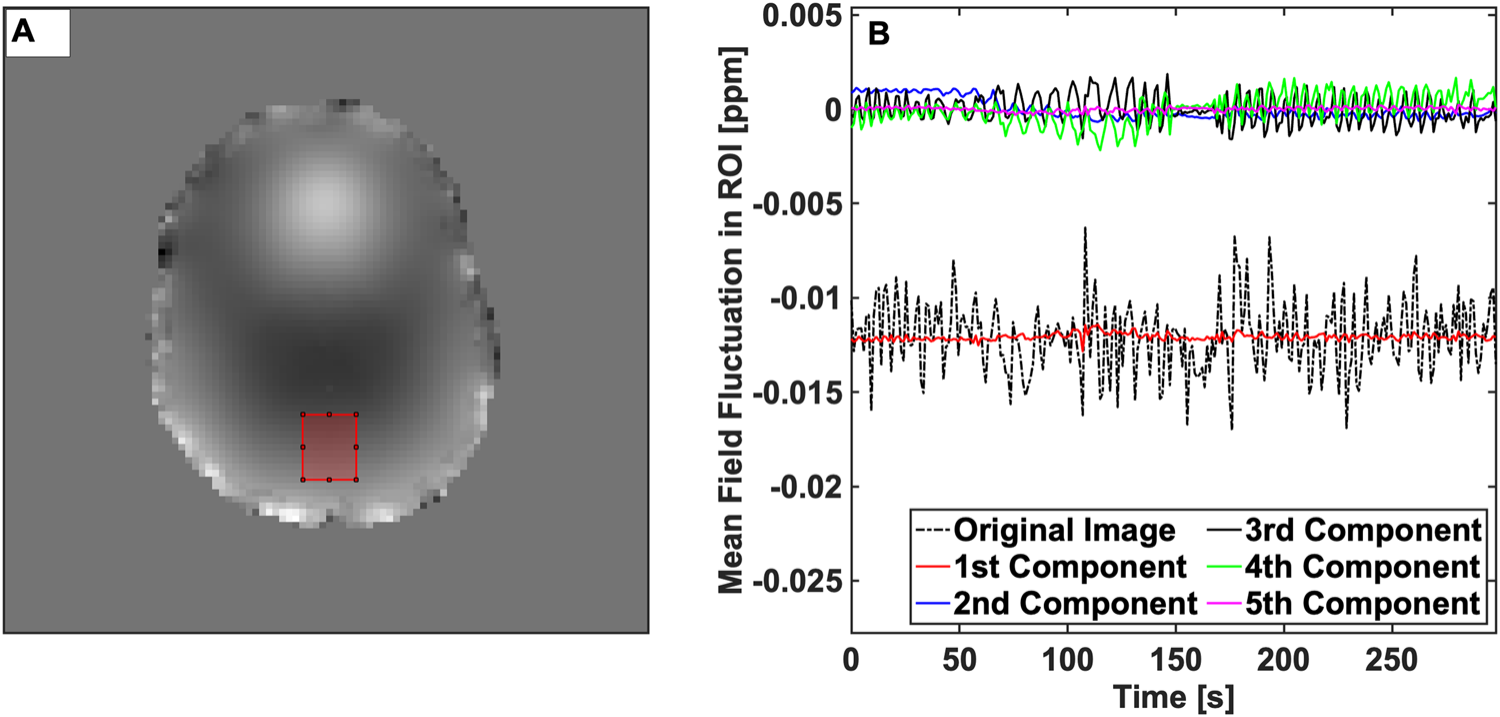
(A) An example region of interest, depicted in red, overlaid onto the mean of the 1^st^ component of the harmonic field decomposition in an example slice from the control subject. (B) Time-varying magnetic field within the region of interest shown as a dashed line. The colored lines show the first 5 right singular vectors in the time domain, which sum to the raw field evolution (dashed). The black (third) component is clearly periodic and contains most of the temporal variation in the measured magnetic field.

Discrimination of the harmonic field component corresponding to respiration ${\tilde{v}}_{r}\left(t\right)$ was performed by finding the scaled right singular vector ${\tilde{v}}_{i}\left(t\right):i\in \left[1:5\right]$ of the low-rank representation $P_{L}$ with maximal instantaneous variation. This was accomplished by defining *${\tilde{v}}_{r}\left(t\right)$* to be the ${\tilde{v}}_{i}\left(t\right)$ for which $\left|\frac{d{\tilde{v}}_{i}\left(t\right)}{dt}\right|$ was maximized. A visualization of the separated harmonic field components is shown for an arbitrary region of interest in Figure 2.

After discrimination of the correct ${\tilde{v}}_{r}\left(t\right)$, the resulting field estimated from ${\tilde{v}}_{r}\left(t\right)$ at each timepoint was projected onto the set of solid harmonics (up to third order) to produce a matrix of solid harmonic coefficients which describes the time evolution of the respiration-induced field modulation. The zeroth coefficient was chosen as the respiratory regressor for comparison with the breathing belt measurement. Pseudocode for the proposed method is given in Algorithm 1. Processing of the data was performed in MATLAB and took 150 seconds on a 32-core workstation.

**Table:**
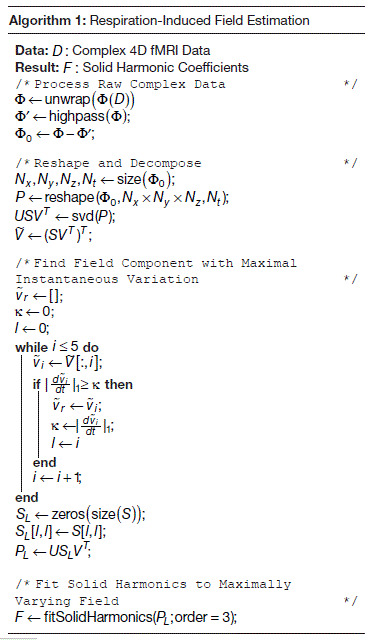


**Algorithm 1:** Pseudocode outlining the proposed method. Variables U and V are the lefthand side and righthand side of the SVD of the measurement matrix P respectively, and S is a diagonal matrix containing the singular values of P on its diagonal. $\tilde{V}$ is a matrix containing the right singular vectors of P scaled by the corresponding singular values.

Processing of the breathing-belt respiratory trace was also performed in MATLAB using standard tools available in the PhysIO toolbox (Kasper et al., 2017). The trace was lowpass filtered and de-spiked to remove spurious noise. The breathing-belt respiratory trace was sampled at ~500 Hz, while the field evolution derived using the proposed method was sampled every volume TR (1150 ms, or 0.87 Hz). An offset correction step was performed to ensure that recorded sampling times for both measurements were synchronized. The temporal evolution of the field modulations measured using the proposed method was compared to the breathing-belt respiratory trace by measuring the error in respiratory period and peak/trough locations between the signals. Peak locations were identified and compared across both traces, and overlapping peaks were identified as those present in both traces within a time interval of half of the volume TR (575 ms). As well, visual comparison of the respiratory trace and the field modulations was performed for all subjects, and the timepoints associated with breathing pattern changes were observed and compared with the instructions given to the control subject.

Synthesis of respiratory phase was performed for the control subject using a histogram-based method proposed for RETROICOR, as well as a Hilbert transform method, and applied to both the zeroth order field modulation and the respiratory sensor signal (Glover et al., 2000; Harrison et al., 2021; Noto et al., 2018). The reference respiratory phase and the respiratory phase obtained from the proposed method were compared by measuring the error in respiratory period and peak/trough locations.

## 3 Results

**Fig. 3.**
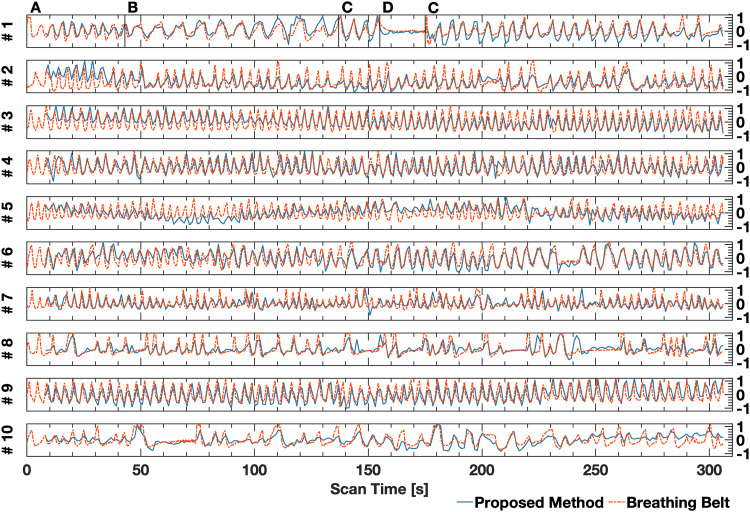
Respiration induced zeroth order field modulation derived from fMRI time-series data using the proposed method (blue) and externally monitored respiratory trace (red, dashed) for all fMRI scans (numbered #1-10). Strong agreement is shown between both the proposed method and the externally monitored respiratory trace. Each trace depicted (for both methods) was normalized to the interval [-1, 1] to facilitate visual comparison. The temporal range and sampling interval (x-axis) were identical for all fMRI scans. Controlled breathing intervals are indicated for subject #1 using the letters A, B, C, and D. Label A denotes shallow breathing, label B denotes deep breathing, label C denotes free-breathing, and label D denotes a breath hold. Breathing was unregulated for the other 9 scans.

Measured field modulations correlated strongly with the respiratory trace obtained from the breathing belt for all subjects. Correspondence with the reference respiratory phase was maintained across shallow, deep, free-breathing, and breath-hold periods, and for the range of respiratory frequencies observed across the subject cohort (Fig. 3). Peaks of the respiration-induced field modulation were well-resolved, with no obvious aliasing in the reconstructed coefficient vectors. For the control subject (Fig. 3, #1), distinct periods of controlled breathing were seen that corresponded directly with subject instruction. The root mean-square error in the measured respiratory period was 0.3 seconds between the ground-truth and proposed method for the control subject.

The overlap in detected peaks across trace obtained with the proposed method and the breathing belt trace was 94% over all scans. Across all subjects, the root mean-square error in measured respiratory period between the ground-truth and the proposed method was 0.68 seconds or 17% of the average respiratory period. The mean error in peak locations between the ground-truth and proposed method was 0.57 seconds. The mean of the respiratory period measured across all subjects using the proposed method was 4.79 seconds ± 1.6 seconds, and the mean measured with the breathing belt was 4.79 seconds ± 1.6 seconds.

**Fig. 4.**
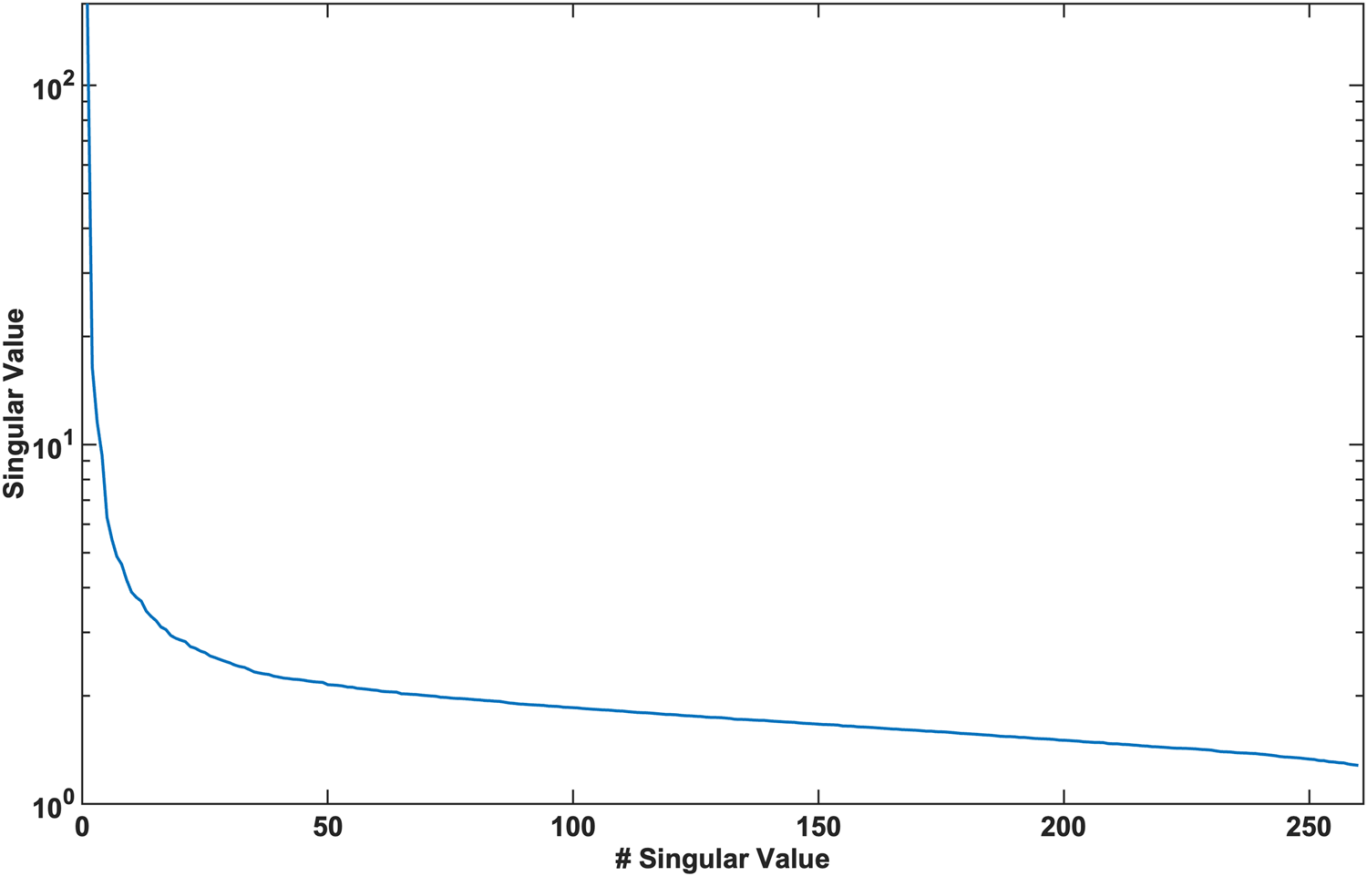
Singular values of the matrix P demonstrating significant decay beyond the first 5 singular values. The first 5 singular vectors describe 97% of the variance in the data, validating the assumption in the proposed work that the matrix P can be well-characterized by a low-rank approximation.

The spectrum of singular values (Fig. 4) obtained from the SVD of P was consistent with the assumption that fluctuations in the harmonic field were low rank, indicating that $P_{L}$ was a good approximation of P. Isolation of the ${\tilde{v}}_{i}\left(t\right)$ corresponding to respiration was reliably performed using our method. The L_1_-norm distribution of the derivatives of the columns of $\tilde{V}$ (Algorithm 1) was found to be consistently dominated by one component for all subjects and was not biased towards the component associated with the largest singular value of P.

**Fig. 5.**
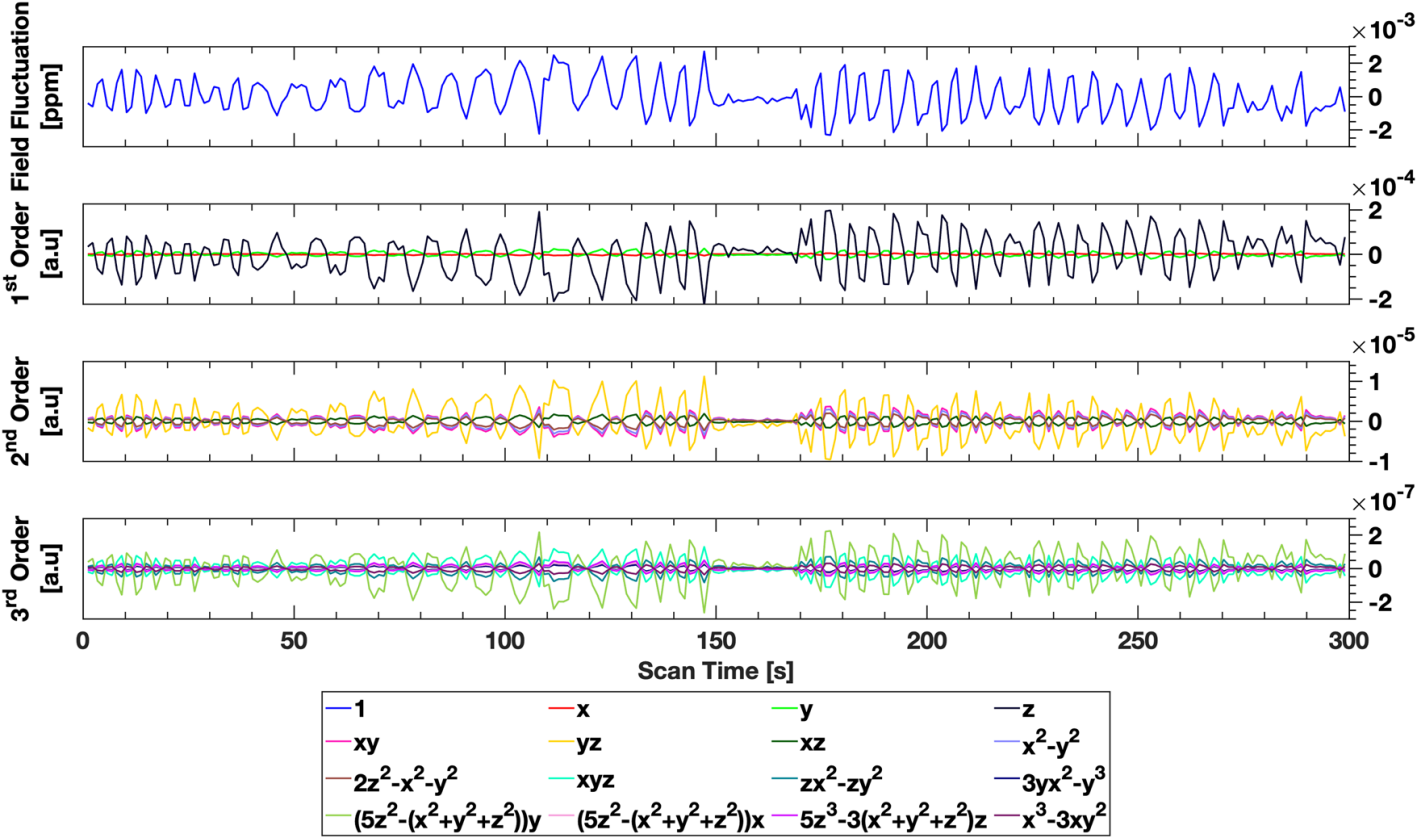
Projection of the temporal evolution of the field onto solid harmonics (see legend for definition) for the control subject, obtained using the proposed method. The primary fluctuation observed is in the zeroth order coefficient, however strong correlations are seen across all coefficients, indicating that respiratory induced field fluctuations can likely be described by a single mode. Instructed breathing periods are seen in all coefficients. The zeroth order fluctuations demonstrate a maximal field excursion of 2.5 ppb, however local field excursions due to higher order coefficients can be up to 15 ppb.

Determination of the solid harmonic coefficients describing the temporal and spatial behavior of the respiration-induced field during respiration was possible in all subjects, and the solid harmonic fitting procedure did not result in significant spurious coefficient values across the whole scan duration. This is seen in Figure 5, where the patterns of controlled breathing are evident in all solid harmonic basis function coefficients.

**Fig. 6.**
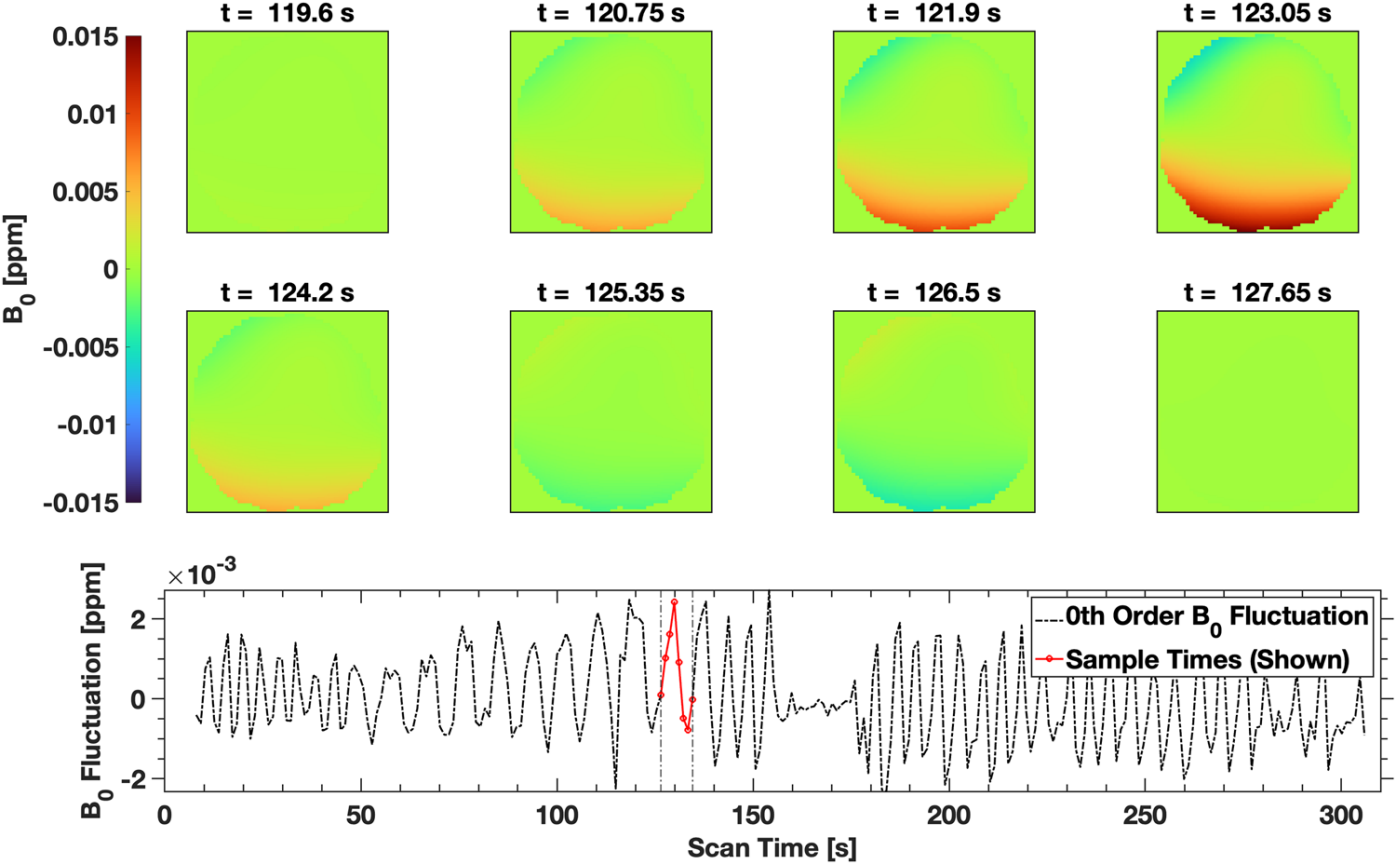
Illustration of the respiration-induced field modulation during a single respiratory period for the control subject. Each image is a masked depiction of the field in the central axial slice of the imaging volume, sampled during the scan at time points indicated in the lower sub-figure. The maximal field excursion over the respiratory period in the imaging volume is [-5 ppb, +15 ppb].

Maximal field excursions due to respiration were observed to be between -5 ppb and +15 ppb within the imaged volume for the control subject, which corresponds to an approximate range of 2.2 Hz at 3 T, consistent with previous measurement of field perturbations due to breathing (Van de Moortele et al., 2002; Vannesjo et al., 2018). The average maximal field excursion over all subjects within the imaging volume was 3.05 Hz ± 1.2 Hz. These excursions were most significant in the periphery of the imaging volume, where the higher order terms in the solid harmonic expansion become dominant, as seen in Figure 6.

**Fig. 7.**
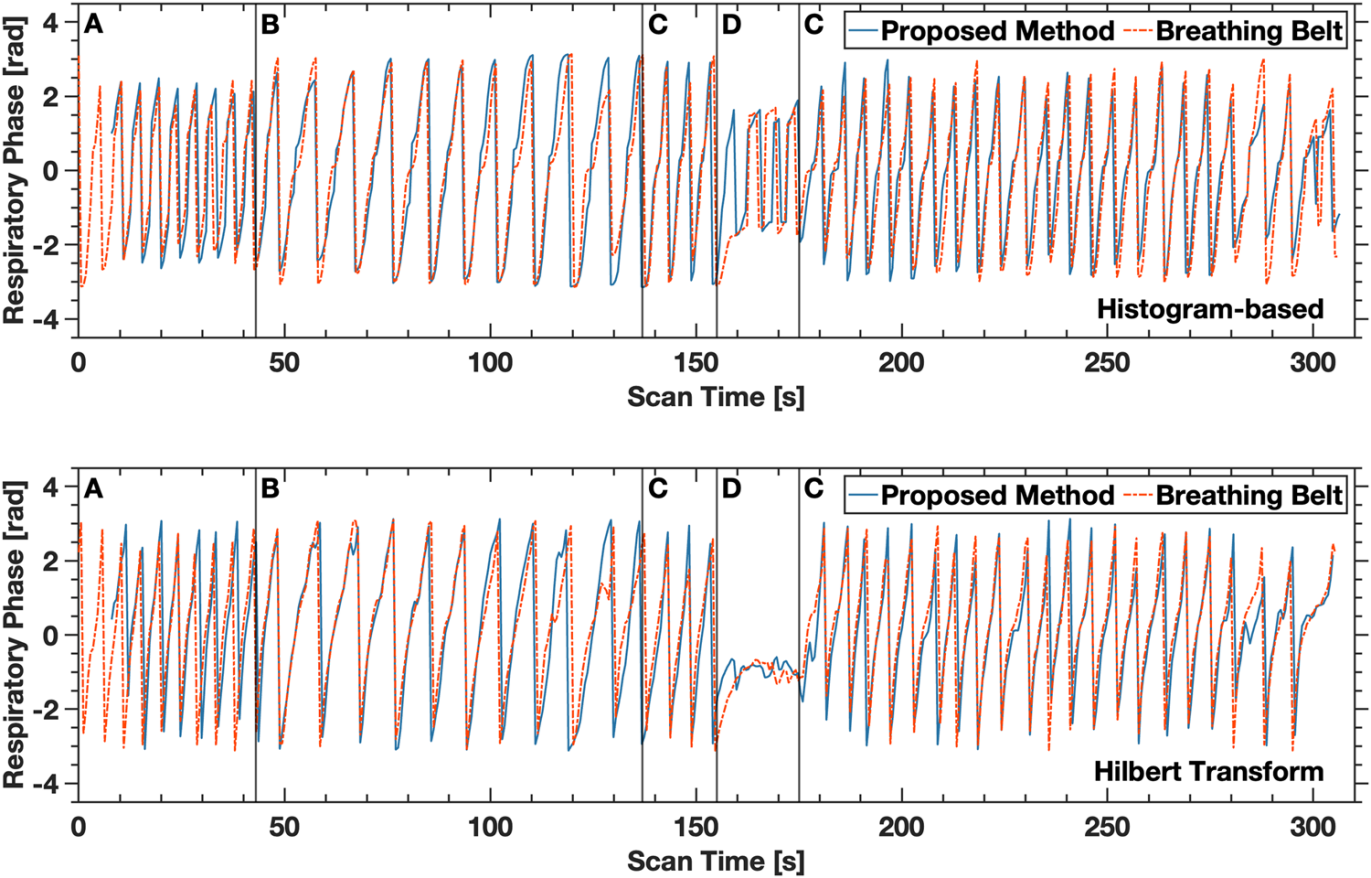
Respiratory phase derived from fMRI time-series data (blue) and externally monitored respiratory phase (dashed red). The top image was generated using a histogram-based respiratory phase calculation method, and the bottom image was generated from instantaneous phase calculated using the Hilbert transform. Structured breathing patterns are denoted with the letters A through D. Shallow breathing is denoted with the letter A. Deep, slow breathing is denoted with the letter B. Free breathing is denoted with the letter C. A breath hold was conducted during the period denoted with the letter D. Strong agreement is shown between both the fMRI-derived respiratory phase and the externally monitored respiratory phase, with peak and trough locations showing good correspondence.

Synthesized respiratory phase from the zeroth order solid harmonic fluctuations agreed strongly with the respiratory phase obtained from the breathing belt (Fig. 7). Distinct periods of controlled breathing were seen in both synthesized and reference traces that corresponded directly with subject instruction. Peaks of the respiratory phase were well-resolved, with no obvious aliasing in the reconstructed signal. For the control subject, root mean square error between respiratory period measured with the breathing belt and derived from the fMRI acquisition was 0.31 seconds. Correspondence with the reference respiratory phase was maintained across shallow, deep, and free-breathing periods. Respiratory phase was not accurately determined during the breath-hold period using either the proposed method or the physiology sensor.

## 4 Discussion and Conclusion

We demonstrate the feasibility of hardware-free estimation of respiration-induced field modulations from fMRI image data on a cohort of 7 healthy volunteers. The proposed method is concurrent with the fMRI experiment, requiring no additional or interleaved reference or navigator acquisitions. The method can be applied retrospectively if the image phase is available and facilitates physiological noise correction by providing an independent respiratory regressor in fMRI studies for which external physiology monitoring is not available or corrupted (Birn et al., 2006; Golestani & Chen, 2020; Power et al., 2020).

The proposed method was shown to be applicable to subjects with a wide range of respiratory rates, as seen in Figure 3. However, an important limitation is that the TR must be shorter than half of the shortest respiration period, a necessary condition for alias-free respiratory trace reconstruction. While this limitation was not encountered in the scope of the present work, the proposed method would not be applicable to populations for whom the resting respiratory rate is greater than twice the volume acquisition rate (1/TR). This limitation also indicates that the proposed method cannot be used to measure cardiac pulsation, which is normally an order of magnitude higher in frequency than respiration. Nevertheless, there is significant effort in fMRI research to increase temporal resolution with novel image acquisition and reconstruction strategies, such as 3D spiral readouts for fMRI (Kasper et al., 2022; Moeller et al., 2010; Engel et al., 2021; Narsude et al., 2014, 2016). These strategies have been demonstrated in vivo with a TR as short as 200 ms for the whole brain volume, which is sufficiently short to adequately sample respiratory fluctuations even in populations with very high respiratory frequency, such as newborns or very young children.

Obtaining breathing information using the fMRI data has several advantages over recording respiration with a belt. Using the belt is prone to errors, for example in obese or small participants. In situations of low chest movement, for example in the case of abdominal breathing, respiration may not be adequately detected. Furthermore, in some populations, the belt may be distracting and wearing it may interfere with the fMRI experiment. The breathing information obtained with the proposed method is automatically synchronized with the fMRI experiment and the processing of the data requires no user interaction. The measured error in respiratory period between the ground-truth belt recordings and the proposed method is less than the volume TR of the fMRI scan, and respiratory period group mean and error are identical across both methods. If the fMRI data are usable for fMRI analysis, they are automatically usable for the tracking of breathing, provide the phase information is saved.

The impact of respiratory period measurement error on physiological noise removal performance has not been specifically studied at the timescale corresponding to the 17% (680 ms) error reported for the proposed method, however there is significant literature on the impact of regressor shifts on fMRI analysis. Birn et al. describe a method by which optimally delayed respiratory regressors can be found for each voxel (Birn et al., 2006). The regressor is shifted with respect to the fMRI signal by -10 seconds to +15 seconds, and while an optimal shift does exist for each voxel, strong correlations were found to be relatively insensitive to shifts of a few seconds (Birn et al., 2006). As well, the respiration response function (RRF), which models the response of the fMRI signal in the brain to respiratory changes, has a duration of 25-40 seconds and varies across different regions of the brain (Birn et al., 2008). Given that changes in fMRI signal with respiration are the result of a convolution with the broad RRF, the accuracy of the proposed method is unlikely to be a limiting factor in resting-state analysis. This likely holds for task-based fMRI as well, as the haemodynamic response function (HRF) is 5-7 seconds in duration (Buxton et al., 2004).

The proposed method is physics driven, using the phase of the acquired fMRI images as the input data. A drawback is that it cannot be applied retrospectively to existing fMRI scans if phase information is unavailable. In such situations, estimation of breathing using approaches that work with the magnitude data have been proposed as a solution, including machine learning (ML) approaches such as a neural network, or deep learning (DL) methods (Addeh et al., 2023; Salas et al., 2020). The training of such approaches requires considerable quality assurance and data cleaning by hand, such as the removal of spike artifacts in the breathing belt data. Only about 14% of the data from the Developing Human Connectome Project could be used for training (Addeh et al., 2023; Somerville et al., 2018). In addition, the same authors identified 52.1% of the Young Adult HCP and 18.9% of the Aging HCP breathing data as good quality respiratory signals (Harms et al., 2018; Smith et al., 2013). Power et al. report that any of three measures typically derived from breathing belt data (respiration variation, the envelope of the respiratory trace, and respiratory volume per time) may miss deep breaths (Power et al., 2020). These findings further underline the notion that field fluctuations may be a more reliable measure of breathing than using a belt, especially in pediatric and aging populations. Retrospective application of ML or DL approaches may require the acquisition of additional training data to tune the network to the specific parameters of the fMRI scan. Furthermore, ML or DL approaches which are applied to magnitude data must extract a respiratory regressor from the BOLD time-series, which is the signal of interest in fMRI. This presents the potential for the respiratory regressor to attribute true BOLD contrast fluctuations to respiration, which would be incorrectly removed upon physiological noise correction. While training on a large, augmented dataset can improve generalizability of ML or DL methods, the acquisition of such a dataset can be difficult for some populations, and the possibility of confounding respiratory and true fMRI signals cannot be eliminated.

Higher temporal resolution field estimation has been reported, with a significant compromise with regards to imaging resolution (Zahneisen et al., 2014). It is, in principle, feasible to extend the proposed method to take advantage of the slice groups acquired in simultaneous multi-slice encoding schemes. This allows for the fitting of solid harmonics at the repetition time of each slice group (slice group TR), presenting an increase in temporal sampling rate by at least an order of magnitude (a factor of 19 in the protocol used in the present work). Fitting to the slice group TR was investigated, however it was not found to yield significant improvements over fitting to the volume TR. Further refinement to the multi-slice parallel imaging strategy would likely be needed to achieve different results, for example increasing the number of slice groups and reducing the gap between simultaneously acquired slices.

The choice of basis expansion to describe the field changes during fMRI acquisition was chosen as the set of solid harmonics up to 3^rd^ order, consistent with existing field estimation work, however use of the proposed method for respiratory phase synthesis would only need an expansion of 1^st^ order. The proposed method is fundamentally similar to a physiological noise measurement approach which uses the phase evolution of external field probes to measure harmonic field perturbations (Gross et al., 2017). Such a technique allows for estimation of the same solid harmonic field expansion as the proposed method, and is capable of measurements concurrent with the imaging experiment, albeit at a much higher temporal resolution than the volume or slice TR. The field probe approach has been shown to be accurate and amenable to fMRI sequences, and thus could serve as a reference measurement for further validation of the proposed method.

Global changes in blood oxygenation, for example due to the inhalation of CO2, can result in field changes of zeroth order. The susceptibility difference between fully oxygenated and fully deoxygenated blood is 264 ppb (Spees et al., 2001). Breathing of 5% CO2 changes venous blood oxygenation from ~0.5 to ~0.7 (Sedlacik et al., 2008). The venous volume fraction in the brain is ~1.7 to 2.5%, because around two thirds of total blood volume resides in veins (Leenders et al., 1990). Then, the global change in susceptibility due to the inhalation of 5% CO2 is between 0.75 and 1.5 ppb, which is about an order of magnitude smaller than the zeroth solid harmonic term.

Recently, a method for physiological regressor estimation on a slice-TR timescale has been proposed (PREPAIR), which identifies time-series signals from magnitude and phase of the fMRI images and performs power spectrum analysis and filtering to derive both respiratory and cardiac regressors (Bancelin et al., 2023). While this method utilizes the phase of the reconstructed image to aid regressor estimation, it does not comprise estimation of the magnetic field changes due to respiration but uses the phase as a source of physiological noise information. While it has been demonstrated as a robust method for regressor estimation and is notably capable of extracting a cardiac regressor sampled at slice-TR, it does not produce an estimate of the magnetic field during the fMRI acquisition. Furthermore, the regressors obtained from PREPAIR are determined to be independent of the BOLD signal by a filtering process in the frequency domain, whereas in the proposed method the estimated respiratory regressor does not require frequency domain filtering.

The computational overhead of the proposed method is minimal, only requiring the phase to be saved at the console and exported to a standard imaging format such as DICOM. The phase must be reconstructed regardless of whether it is exported, so no additional reconstruction time is required during the imaging session. Processing of the exported phase images can be done offline in ~2 minutes for the whole fMRI time series of 260 volumes (Kames et al., 2018). Standard processing pipelines for fMRI data, such as fMRIprep, already have significant computational overhead, and adding the proposed method as an additional step would not be a disruption to fMRI workflows (Esteban et al., 2019). The proposed approach is not limited to fMRI, but can be employed in all scans that acquire gradient echo data at sufficiently high temporal resolution, such as dynamic susceptibility contrast MRI or measurements of cerebrovascular reactivity (Ostergaard, Sorensen, et al., 1996; Ostergaard, Weisskoff, et al., 1996; Fisher et al., 2018). In conclusion, the phase of the fMRI scan is sensitive to subject breathing and can be used for the tracking of respiration, without the need for additional hardware, nor the recording of additional data.

## Data and Code Availability Statement

The current implementation of the proposed method is available in MATLAB. Implementations of the phase processing algorithms used in this work are part of the open-source QSM toolboxes and are available in MATLAB and Julia programming languages (https://github.com/kamesy/QSM.jl, https://github.com/kamesy/QSM.m). The code to reproduce the results from this paper is available at https://github.com/alexjaffray/fMRI-phase-toolbox and the data is available upon request.
